# Resilience in Agriculture: Communication and Energy Infrastructure Dependencies of German Farmers

**DOI:** 10.1007/s13753-022-00404-7

**Published:** 2022-03-19

**Authors:** Franz Kuntke, Sebastian Linsner, Enno Steinbrink, Jonas Franken, Christian Reuter

**Affiliations:** Science and Technology for Peace and Security (PEASEC), Technical University of Darmstadt, 64289 Darmstadt, Hesse Germany

**Keywords:** Digitalization of agriculture, Germany, Infrastructure failures, Resilience capacities

## Abstract

Agriculture is subject to high demands regarding resilience as it is an essential component of the food production chain. In the agricultural sector, there is an increasing usage of digital tools that rely on communication and energy infrastructures. Should disruption occur, such strengthened dependencies on other infrastructures increase the probability of ripple effects. Thus, there is a need to analyze the resilience of the agricultural sector with a specific focus on the effects of digitalization. This study works out resilience capacities of the interconnected technologies used in farm systems based on the experiences and opinions of farmers. Information was gathered through focus group interviews with farmers (*N* = 52) and a survey with participants from the agricultural sector (*N* = 118). In particular, the focus is put on the digital tools and other information and communication technologies they use. Based on a definition of resilience capacities, we evaluate resilience regarding energy and communication demands in various types of farm systems. Especially important are the resilience aspects of modern systems’ digital communication as well as the poorly developed and nonresilient network infrastructure in rural areas that contrast with the claim for a resilient agriculture. The result is a low robustness capacity, as our analysis concludes with the risk of food production losses.

## Introduction

Digitalization, especially the interconnection of modern equipment for agricultural production, is a major issue in the agricultural sector. The desired positive aspects are an increase in efficiency and effectiveness and a more resource-friendly production of food. Digital, interconnected tools could open up new paths to profitable and socially accepted agriculture that benefits the environment, biodiversity, and farmers (Weltzien 2016).

Smart farming tools can be useful in gaining precise information about crop conditions for planning farming practices according to specific phenological stages and thus improving, for instance, the timing for harvest, pest control, and yield protection (Yalcin 2017; Braun et al. 2018). But the increasing usage of digital solutions may also increase agriculture’s dependence on digital infrastructures. Current resilience assessments (Meuwissen et al. 2019; Perrin and Martin 2021; Snow et al. 2021) for agriculture lack a specific view about the digitalization of agricultural systems and the possible consequences of its interactions with other key systems like the energy and communication sectors. Unintended consequences could be new risks and threats to the business of farms and—from a global perspective—also to food safety. As Darnhofer (2021, p. 3) states: “While much research has focused on developing efficient processes and increasing productivity, much less research effort has gone into understanding what enables agricultural systems to navigate unexpected change.” Going further within the research area of food system resilience, we will investigate organizational behavior in (technical) emergencies as well as the digitalization process from a farmer’s point of view, and draw conclusions about potential risks in relation to the current digitalization process, using the situation in Germany as an example for modern, technology-driven agriculture. This study is guided by the following research questions:

- RQ1: What potential risks are associated with the use of digital tools, especially related to infrastructure failures for farm systems?
- RQ2: What are the resilience capacities associated with the use of digital tools of agricultural companies?

The remainder of this article is structured as follows: In Sect. 2, we present the definition of resilience used for this study as well as the problems and vulnerabilities associated with modern agricultural technology, for example, smart farming tools. Section 3 describes the focus groups we conducted, in which a total of 52 farmers participated. In order to verify our preliminary findings of the qualitative study, we conducted a quantitative survey on specific topics with 118 agricultural experts, which is described in Sect. 4. Subsequently, in Sect. 5, we contribute to the existing literature by relating the answers of both the qualitative and quantitative studies to the definition of resilience and to the digitalization process in agriculture. Finally, in Sect. 6, we conclude with a summary of key findings and set out possible future research.

## State of Research

In order to meet the social responsibility of agriculture as critical infrastructure (CI), digitalization through the incorporation of new technologies in agriculture has become a major issue. We present the current state of digitalization as well as already known risks coming along with the increased usage of information systems. We also present a framework for resilience assessment of farm systems and derive a research gap.

### Interconnected Technology in Agriculture

Scrutinizing the current literature, various obstacles and problems as well as current trends regarding the effective and efficient use of digital, interconnected technology in farming business can be identified. First, the use of communication tools is changing. Communication is of great importance in the daily work routine of a farmer. Arrangements with employees or external contacts, such as suppliers, subcontractors, or clients, are increasingly incorporated into new technologies. A study by von Hobe and collaborators (Hobe et al. 2019) investigates the ways farmers communicate and expected developments between 2017 and 2022. According to their results, in 2017 only 15% of German farmers considered digital services (email, messenger, and cloud services) as their most important means of communication for their everyday work routine, while 36% anticipated digital services as their principal means of communication in 2022. Braun et al. (2018) state that in the agricultural sector, smooth communication and information exchange along the supply chain is essential; only limited attention has been drawn to this topic. Shang et al. (2021) propose a framework for modeling adoption and diffusion of digital farming technologies based on reviewed literature. As the authors state “only a few recent studies highlight the importance of attributes of technology (e.g. compatibility to existing farming equipment, complexity and data safety)” (Shang et al. 2021, p. 12).

Another important issue is the demand for Internet connectivity. The analysis of wireless sensor networks for precision agriculture by Jawad et al. (2017) discovers that current approaches typically propagate an Internet connection to cloud services as a means to analyze sensor data and provide access via client computing devices, such as tablets. At the same time, technologies like cloud services have proven vulnerable to threats such as cloud-service breakdowns or Internet outages (Aceto et al. 2018). Moreover, the digital infrastructure in Germany is characterized by a digital divide, which means that rural areas have less access to high-bandwidth Internet connections than urban areas; for example, 4G networks provide 73.5% coverage in rural areas, 82.2% in urban areas (Rizzato 2019). Faults in interconnected technology may be caused by several factors. For a first overview, it is useful to distinguish between information and communication technology (ICT) related and energy-related failures.

*ICT-related incidents*: In general, there are many different causes of ICT disruptions that are not energy-related. Aceto et al. (2018) offer a categorization that evaluates incidents on three axes: origin (natural/human), intentionality (accidentally/intentional), and type of disruption (physical/purely logical). An example of ICT outages caused by natural circumstances that result in the physical damage of ICT infrastructure is the fiber cable cut by undersea currents that put the Commonwealth of the Northern Mariana Islands offline for three weeks in 2015 (SubCableWorld 2015; Aceto et al. 2018). As far as human-caused failures are concerned, there are cases of intentionally caused ICT outages. The Internet shutdown in Egypt and Libya in 2011, for example, was carried out by the government for censorship reasons (Dainotti et al. 2011), as were ransomware or distributed denial-of-service (DDoS) attacks that resulted in large cyber crises in the UK in 2017 and Estonia in 2007 (Backman 2021). A common example that accounts for the majority of Internet backbone disruptions is the accidental damage to submarine cables through fishing activities and dragged anchors (CCDCOE 2019). Purely logical disruptions, like prefix hijacking, can also lead to major constraints with regard to the accessibility of a web service (Ballani et al. 2007). Even on the level of farms, network technology like wireless sensor networks could be attacked (Kuntke et al. 2022).

*Energy-related incidents*: On many farms, especially in livestock farming, certain facilities (like ventilation) depend on a permanent power supply. Power outages can therefore result in the loss of an entire livestock population. Storms and floods are the most prevalent causes of major power outages in Central Europe (Mahdavian et al. 2020). For example, during a three-day power outage after a blizzard in northwest Germany in 2005, most of the farmers had to secure their livestock facilities through generators (Gerhold et al. 2019). In some cases, livestock farms without functioning backup energy supply lost all their livestock within hours of disconnection (Schröder and Klaue 2005; Pfohl 2014), as weather conditions prevented taking animals outside and multiple critical situations at different locations at the same time required triage. Obviously, most of the interconnected technology also depends on electrical energy. While some of these systems, such as outdoor sensors, could run on battery, information technology (IT) hardware, monitoring of a ventilation system, or smart valves could require a stationary power source. The increasing reality of cyber hazards, and the related need for systems to become more resilient, has pushed CI protection and resilience in the information system sector up the agendas across a range of domains.

### Resilience Assessment of Farm Systems

In this article, we focus on the impact of digital technology for farm systems on resilience. The term resilience varies depending on the context of application, such as engineering (Francis and Bekera 2014) or ecology (Holling 1973), which is why a universal definition is not possible. According to Tendall et al. (2015, p. 18), for food systems “resilience can be broadly defined as the dynamic capacity to continue to achieve goals despite disturbances and shocks.”

To assess the resilience of a system, however, a suitable framework is needed. A contribution by the European SURE-Farm project highlights the complexity of agricultural systems and states that previous studies mostly focused on agricultural production processes (Meuwissen et al. 2019). The authors therefore propose a resilience assessment framework that incorporates not just agricultural production properties, but also farm demographics and governance. Even more important for this paper, the authors define three resilience capacities, which are needed to evaluate a systems’ resilience: robustness, adaptability, and transformability. According to Meuwissen et al. (2019, p. 4) robustness is the resilience capacity “to withstand stresses and (un)anticipated shocks” [sic]; adaptability is the capacity that allows a system to adjust to undesirable situations by undergoing some changes, but without changing the internal structures; transformability represents the capacity to significantly change internal structures to return to normal or improved operations.

We understand resilience not only as crisis management but also as a preventive and foreseeing concept. The goal of resilience is the regular continuity of the system under every circumstance. This is especially important when taking into consideration the characterization of farm systems as CI.

### Research Gap

Nearly all of the scientific literature on ICT for agriculture is concerned with improving agricultural operations through greater automation or increased precision—see, for example, Gu and Jing (2011) and Wolfert et al. (2017). Few works describe dangers resulting from increased dependency on other infrastructures like electricity generation and telecommunication (Moteff et al. 2003; Reuter et al. 2019), which are very important to consider in light of possible disaster situations. Against the backdrop of agriculture being CI and the increasing use of smart farming technologies, the investigation of IT resilience in the context of agriculture seems to be even more crucial. Gurschler et al. (2017) point out that the usage of ICT in CI creates new risks and threats for the IT infrastructure, and for that reason, it is important to engage all actors in proper risk assessments.

Beyond agriculture, providers of other CI also should be aware of their responsibility and possible precautions for potential risk scenarios (O’Rourke 2007; Rademacher 2013). This is essential in order to build a shared understanding of disasters and to comprehensively evaluate past incidents (Monteil et al. 2020). Existing case studies (Meuwissen et al. 2019) or other recent works (Perrin and Martin 2021; Snow et al. 2021) do not cover our work’s technology-driven subject of study, but rather focus on aspects of socioeconomic resilience. In contrast, we focus on the impact of the increasing usage of digital tools on the resilience of agriculture. To our knowledge there are currently no empirical studies that address this aspect of resilience and the preparation for disasters in the sector of agriculture in general and especially for agricultural IT systems.

## Qualitative Analysis

In this section we document the methodological procedure within our qualitative study. To answer our research questions (see Sect. 1), focus group interviews (Kitzinger 1995; Morgan 1997) were conducted with a concentration on resilience in agriculture, especially ICT, and how ICT disruption is considered a possible danger for the regular working procedure on farms.

### Method and Data Description

To avoid (subjective) biases, the focus groups were led by two of our researchers in face-to-face meetings in 2019. The researchers did not have the impression that the participants had hidden agendas or intentionally tried to hide information (Kontio et al. 2004). The researchers conducted 12 focus group interviews (Table 1); these sessions can be considered expert interviews, since all the participants were experts in agriculture. The entire process, which comprised the creation of an interview guideline, recruitment of participants, conducting the focus groups, and data analysis and storage, followed the guidelines of the ethical commission of the Technical University of Darmstadt. Some results of these focus groups have already been published in a scientific journal (Linsner et al. 2021); however, the data were analyzed with a different scope and purpose.

**Table 1:** Distribution of participants of the focus group interviews

Focus group	Participants
fg1 fg2 fg3 fg4 fg5 fg6 fg7 fg8 fg9 fg10 fg11 fg12	P1, P2, P3, P4 P5, P6, P7 P8, P9, P10 P11, P12, P13, P14 P15, P16, P17, P18 P19, P20, P21, P22 P23, P24, P25, P26, P27 P28, P29, P30, P31 P32, P33, P34, P35, P36 P37, P38, P39, P40, P41, P42 P43, P44, P45, P46, P47 P48, P49, P50, P51, P52

#### Participants

Since we are working in publicly funded research projects (HyServ and GeoBox) with partners from the private sector, federal institutions, and associations for farmers, such as machinery rings, their clients and members were invited to participate in our focus groups. Participation was voluntarily and no compensation was paid. Each participant was informed about the objectives and topics of the study and how their answers would be processed via an informed consent form, which was signed by each person. Every participant (*N* = 52) in our study owns or leads an agricultural business. This characteristic has ensured that participants at decision levels were involved. Most of them run farms, but some also are service providers for other farmers. Seven of the participants identified as female and 45 as male. Thus, the proportion of female participants is 13.5%. The latest numbers of the 2016 Eurostat database estimate that the gender ratio of the agricultural workforce in Germany is 32.4% women overall and 9.0% at the operational level of farm management (Eurostat 2016). Hence, the gender proportion of our study depicts the gender ratio of farm managers in Germany closely.

A large percentage (81%, *N* = 42) of the participants are younger than 40 years. While their age group represents less than 15% of German farmers in 2016 (Eurostat 2018), they represent current or future farm managers and therefore are an interesting target group for this study. Our sample covers multiple branches of agriculture, but these are not equally distributed (see Table 2).

**Table 2:** Branches the participants are working in (multiple answers possible)

Branch	Amount
Cultivation of grain	22
Viticulture	3
Cultivation of vegetables	1
Dairy cattle	12
Beef cattle	4
Animal husbandry Pig rearing	4
Laying hens	3
Biogas production	3
Service provision	6

#### Data Collection and Analysis

Data of the focus groups were collected through sound recording followed by transcription. We segmented the data into meaningful expressions using the constant comparison analysis method, which, in its first stage, is characterized by open coding (Onwuegbuzie et al. 2009). These included codes such as: general understanding; IT-risks; IT-prevention; Hardware/PC; Hardware/Tablet, and so on, to name just a few of the 34 codes in total. Subsequently, we formed five categories by grouping the statements/codes: digitalization in agriculture, infrastructure on farms, processes, data protection, and resilience. The research process involved two researchers during both the focus group interviews and the process of coding. They both applied the method of constant comparison analysis independently from each other and compared and consolidated their results afterward. This allowed us to derive our results, as presented in the following section. Because the recorded interviews are in German, we translated the statements into English as literally as possible.

### Results of the Focus Groups

**Fig. 1:**
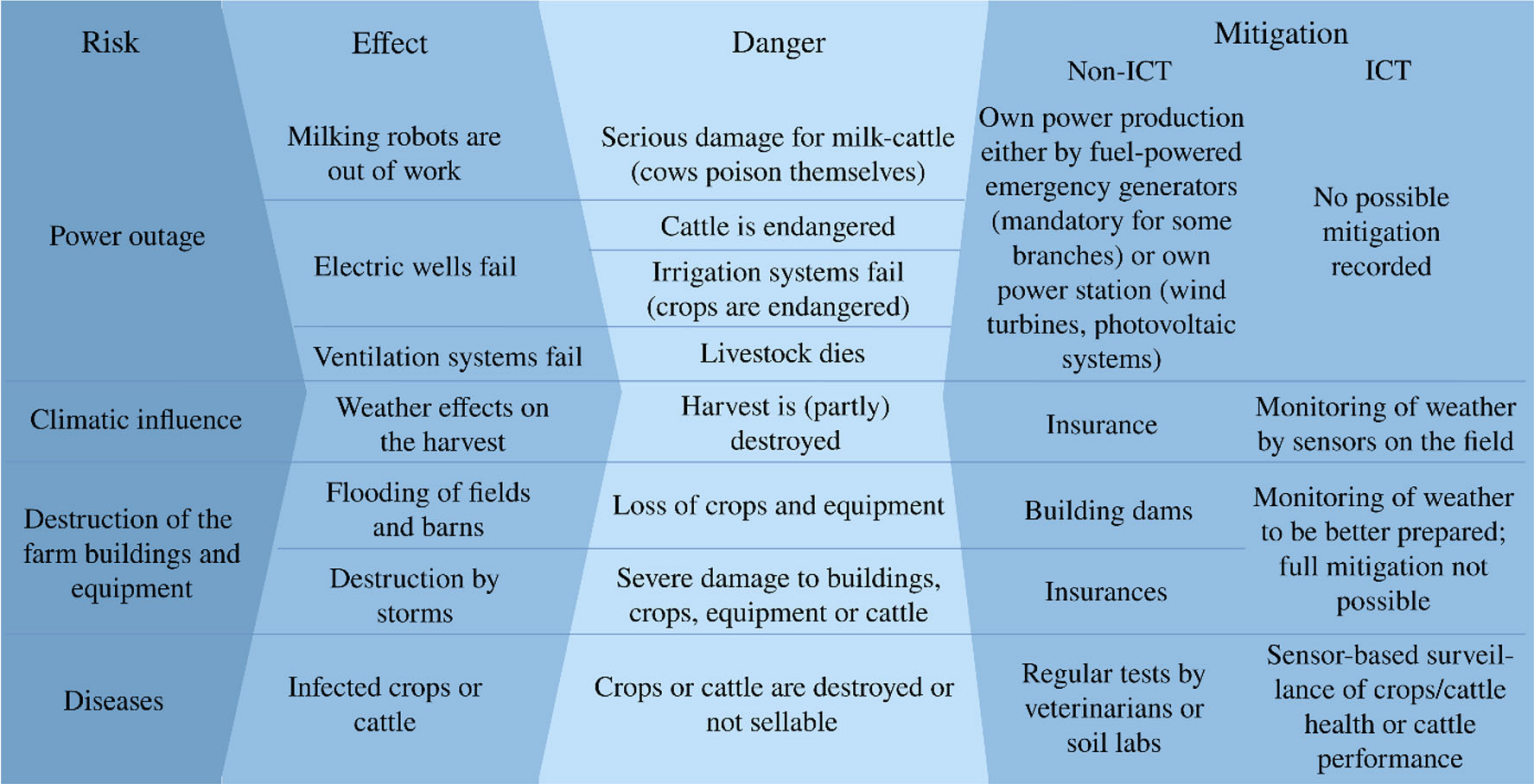
Tabular listing of the recorded risks within the focus groups with possible effects, and the resulting dangers as well as appealed countermeasures, with or without information and communication technology (ICT) to mitigate specific dangers

In this section, we present the results of our interviews, where the constant comparison analysis method led to a grouping of all statements into the following aspects: (1) digitalization in agriculture and (2) resilience. An overview of the insights from the focus groups (Sects. 3.2.1 and 3.2.2) is given in Fig. 1.

#### Digitalization in Agriculture

One central aspect regarding the general understanding of digitalization is summarized in the following statement: “What we used to write on paper is now digital on the PC” (fg5). Other statements mention that digitalization in agriculture contains the use of modern technologies for increased efficiency in conventional working processes as well as the automatic recording of processes on the field (abolishing the unpleasant task of manual recording).

When it comes to an individual’s own experiences and examples, most but not all (7 of 52) of the participants state that they use digitalized hardware in their everyday work processes. As a result, a wide range of experience is represented. Whereas some participants with an already-digitalized work routine use digital tools for direct on-site recording and highly automatized vehicles with digital steering systems and section control, that is, a precise fertilizer application, others in contrast state: “Everything is still handwritten in our case” (fg8). Companies that practice arable farming mostly take advantage of section control, which heavily relies on satellite navigation of the tractors (fg4, fg8, fg10). Some of this hardware already relies on (mobile) network technologies for data exchange: “When I’m at the [fertilizer] injection, I just enter what kind of spray and how much. Then it’s uploaded, and I read it on my computer, and I’m done” (fg11). Regarding livestock farming, there are also robots and corresponding software involved, for example, for monitoring the milk yield and milk quality of each cow. The aggregated data in the case of a dairy farm allows a highly detailed insight, which was commented as follows: “You see everything [...] the ingredients, the amount of milk from each cow [...]” (fg11). Office tasks that have to be performed on farms use common ICTs, such as telephone, computers, fax, or the Internet. Most participants’ companies have a digital crop field card to keep track of all processes on their land. Processes such as application for state subsidies or invoicing as a temporary employment company require operational data of the company, precise recording of work, and transmission of digital forms. Such recording is typically done automatically by modern, digital tools, such as tablet-like tractor additions for recording all processes, “[...] just to be safe in billing” (fg10).

In line with these reported experiences, personal feelings and opinions differ greatly when it comes to digitalization in agriculture. Here, some of the participants see the small size of their company as an obstacle and express opinions like “Do you even need something like that?” (fg10). In contrast, others outline the possibilities, especially for small-sized agricultural companies, as for example: “With small structures, I actually think it makes sense to drive with things like RTK [Real Time Kinematic] or section control [...]. You get [the fertilizer] much better on the target areas” (fg10). Some participants would even like to see (more) digitalized solutions in their companies and describe their current condition of a nondigitalized company as “quite backward” (fg5). There are also some neutral statements, mostly with the central message that digital tools do not seem necessary and that the real benefit is hard to determine at the moment, like: “I think at the moment it is still a bit too early to draw conclusions. [...] In five or 10 years, when the technology is even more mature, and even smaller companies can afford it, things will look different” (fg6). Negative opinions revolve around fears, risks, and problems and are mainly associated with the loss of data sovereignty and related consequences. Some participants have already thought about possible problems that could arise from the exploitation of their business data.

Another fear refers to the increasingly high level of dependence on technology, which, if it fails, can only be repaired by experts: “You are so dependent on the technology. In former times, one could still detect the cause [remark: of technical failures, for example, in machinery] somehow by oneself or could try to get it [agricultural machine] back by oneself. And now there are error messages, and then it’s over” (fg7). This is related to the dissatisfaction about the unreliability of certain products, incompatibility of devices, or frequently crashing software: “And when that [software error] happens several times a day, it’s annoying” (fg7). A similar problem is the incompatibility of iOS and Android applications because some machine vendors only offer useful companion applications exclusively for one of these operating systems (fg4). A further, often mentioned problem is poor education on how to use digital products (fg6, fg7). Especially concerning is data sovereignty; one focus group mentions that it would be great to educate the owners of small-sized farms on how to build up a local Wi-Fi network that is secure and allows sharing certain data with third parties if required. It would be great “[...] if there would be training or enterprises offering a simple intranet or the possibility to just built up Wi-Fi on our farm with a server owned by ourselves, where we store the data and [...], which offers the possibility to share data” (fg9), “We really want to share, in order to process it [the data]. [...] The only problem really is data protection” (fg9). Participants are generally aware of the importance of backups and the security of their computers, but report a lack of necessary knowledge. No participant mentioned the increasing dependencies on other infrastructures, like the Internet or the power grid, as a risk of digitalization, before the interview questions explicitly referred to this subject.

#### Resilience in Agriculture

Regarding the resilience of agricultural businesses, we focus on the domains of electricity and ICT, as we see both dependencies as possible sources for a reduction of resilience. We start with statements about the perceived threats of resilience followed by statements based on the definition of resilience capacities given in Sect. 2.2, which includes (1) robustness, (2) adaptability, and (3) transformability. The following statements are grouped into the domains we perceive to be possible causes for ripple effects—electricity supply and communication and Internet infrastructure:

*Resilience of electricity supply*: Although many agricultural tasks involve tools that need electrical power, some technologies are particularly vulnerable (see Sect. 2.1). In order to (1) cover power outages, power can be self-produced: “We generate electricity via our wind turbine. Also, our whole stable area is equipped with photovoltaics” (fg8). Many participants stated that cattle and pig breeding companies are required by law to have an emergency power generator (including sufficient fuel reserves for its operation) for (2) adaption in power outage scenarios. However, dairy farms that usually use milk robots would have to switch to a manual mode. This could decrease the milk yield, but harm in the form of diseases for their animals would be prevented (fg8). Based on these results, we can see that cattle farmers’ operations are weakened in terms of production quality and efficiency in power outage scenarios. Therefore, we hypothesize that farms that are also active in the livestock sector more often take precautions against outages (H1). Typically, the (3) restoration after a power outage is easy and fast in the domain of agriculture. But for some digitalized tools, a restart could take a long time. After a short power outage in a dairy farm “All the robots and boxes were off, the cows were still inside. So, nothing worked anymore. Until the whole system was started up again and running faultlessly, two days went by” (fg7).

*Resilience of communication and Internet infrastructure*: The necessity of thinking about communication and Internet infrastructure in the form of both landline and mobile radio networks arises because the regular operations of agricultural companies require communication with various actors. Therefore, access to reliable communication infrastructure is essential. Since we assume a rising dependency on various digital communication products, we are interested in the opinion of the participants regarding the current state of reliability and possible dangers and risks in this field. In 2019 (as in 2021), many areas in Germany were still not covered by cellular networks.

Since most of these blind spots happen to be in rural areas, the big majority of the general public is not affected, but farmers are disproportionately impacted. We hypothesize that the incorporated tools of agricultural operations are often dependent on the Internet (*H2*), based on the insight that machine vendors create new products that rely on—or at least take advantage of—a permanent Internet connection, and that this requirement intensifies problems such as missing coverage by cellular networks: “We have technical possibilities to use apps and to do everything. And then it depends on simple things like the bad Internet. [...] I know two years ago that it took me all day to download an application” (fg4). Capacities for absorbing ICT outages appear to be difficult to build up, as there are rarely any suitable mechanisms for this improvement. In fact, no absorbing measure was mentioned by the participants. However, there are some ways to adapt in situations of failing telecommunications. In order to share data between companies, the usage of a USB flash drive is considered sufficient and secure and is not perceived as more demanding than sending it to the tractor directly, although it requires more time for data transport (fg2, fg9). This applies also for the exchange between employees of the same farm or between different devices or for business communication: “Yeah, well, you know where the contractor lives. You can still go there in an emergency and discuss everything with him/her” (fg4). The restoration after a communication and Internet infrastructure outage is seen to be as simple and fast as electricity supply. It was not mentioned as problematic at all, since all the tools reconnect automatically and phones are working again on both sides.

## Quantitative Analysis

Based on survey data, we are able to test hypotheses derived from the qualitative study (Sect. 3.2) and gain more insight into the topic of technological resilience in agriculture. This section elaborates on both descriptive results and hypothesis testing.

### Method and Data Description

Using an online questionnaire, we asked German farmers about resilience in agriculture, dependencies on other infrastructures with a focus on ICT, and potential outage scenarios that could harm their agricultural operations. Initially, this survey was planned to be conducted offline at different agricultural events in 2020. However, these were canceled due to restrictions during the COVID-19 pandemic.

#### Participants and Procedure

**Fig. 2:**
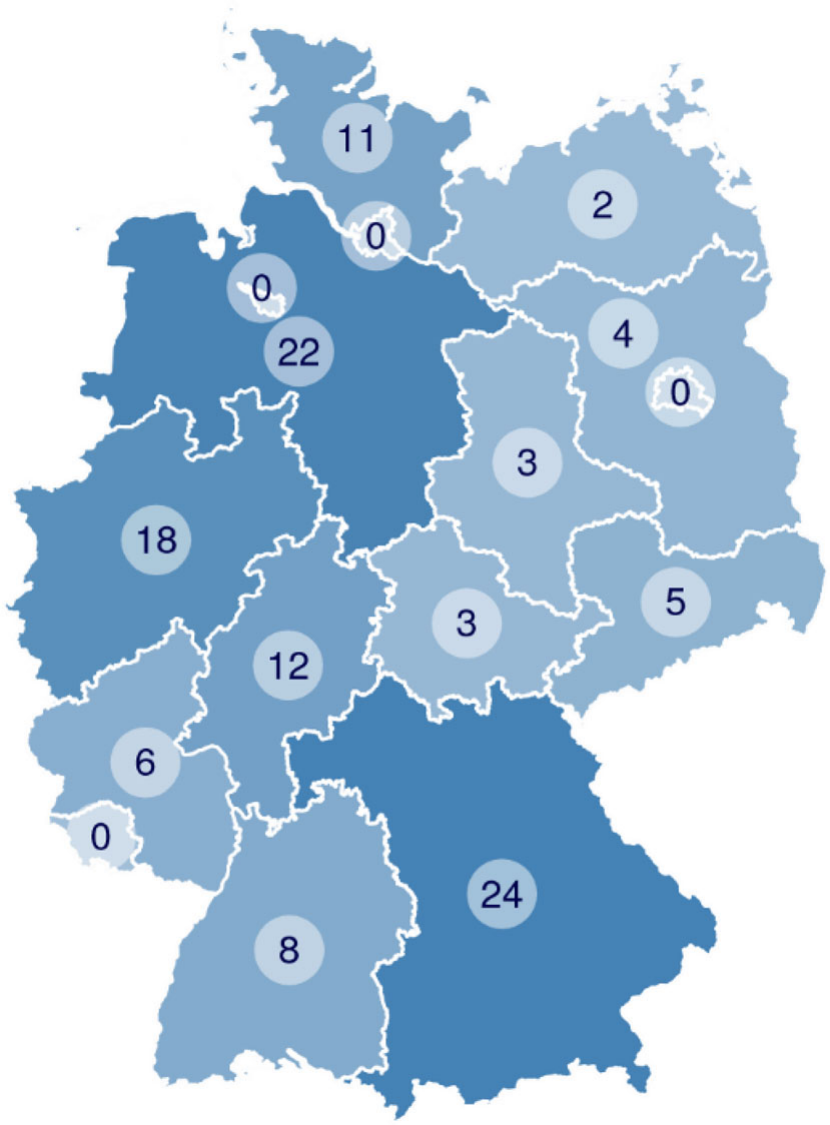
Distribution of the participants of the online survey. We asked for the German federal state of the company’s main activity

The participants were recruited via the mailing list of a German association of farmers. In the survey, we asked for the participants’ specific working conditions. Most of the participants (111) worked as farmers in Germany, and five worked in related business segments, such as food production or agricultural machinery production. Two participants did not specify their employment, but they are also active in the domain of agriculture as recipients of the mailing list. The survey includes farmers working in larger businesses (see Table 3) and covers a more diverse selection regarding age and location than did the focus groups (Sect. 3). Participation was voluntary and not compensated. Within the survey, we collected demographic information and information about the agricultural enterprise in which the participants worked. The age distribution was recorded in categories (see Table 4) with 73% of participants in the age groups 31–60. The geographical distribution of the participants’ work areas in Germany is shown in Fig. 2.

**Table 3:** Companies size in number of employees

Employees (number)	1	2	3	4	5–10	*>*10	N.A.
Relative frequency number	19% 23	35% 41	15% 18	7% 8	11% 13	5% 6	8% 9

**Table 4:** Age distribution of participants

Age (years)	21–30	31–40	41–50	51–60	61–70	*>*70	N.A.
Relative frequency number	15% 18	23% 27	28% 33	22% 26	9% 11	2% 2	1% 1

#### Data Collection and Analysis

For the collection of data, the software LimeSurvey 3.17.0 was used on a self-hosted server. The questionnaire consisted of 50 items with a closed-ended answering scheme. Not all of the questions were relevant for this study. The survey was conducted in German. For the data analysis, only completely filled out questionnaires (*N* = 118) were included. For the statistical evaluation and the graphical visualizations, the authors used R 4.0.2 and the package ggplot2. Because the questionnaire was in German, we translated the items into English as literally as possible.

### Results of the Online Survey

To gain quantitative insights into the (technological) resilience domain of German farmers, we used an exploratory approach in addition to hypothesis testing to evaluate interesting and relevant aspects. Since the online survey also included items on other topics, we have limited ourselves here to the areas of technical resilience and digitalization. We split the presentation of results into two categories: (1) incidents and precautions and (2) estimation of operability. Within these categories, we investigate assumptions we have gained from analyzing the focus groups’ statements (Sect. 3.2).

#### Incidents and Precautions

**Fig. 3:**
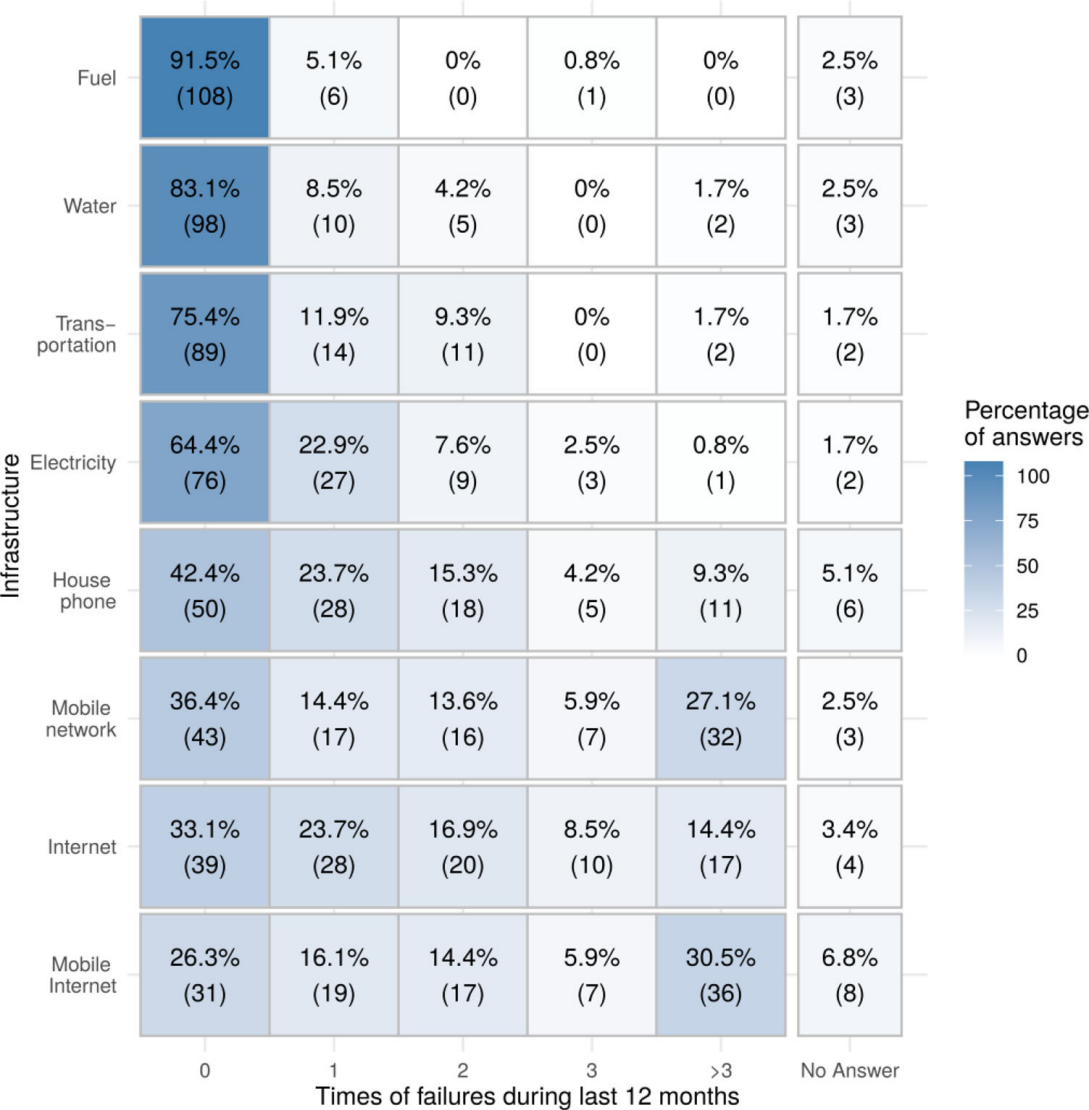
Heat map for failures within the last 12 months depending on infrastructure type. The numbers represent the percentages of the answering options and add up to 100% per row. The numbers in brackets represent the absolute numbers of answers

We asked the participants to give numbers regarding actual outages. As depicted in Fig. 3, especially ICT-related incidents were present in the previous 12 months of the survey, with a quite high proportion of failures of mobile network (cellular phones) and mobile Internet: Participants reported failures of mobile network (27.1%) and mobile Internet (31.5%) more than three times within this time frame. Classical infrastructures like transportation, water, and gas were relatively stable within the same timeframe. Electricity—which is especially important according to the focus groups—was attributed to have caused some outages, with more than 30% having at least one power outage in the past 12 months.

**Fig. 4:**
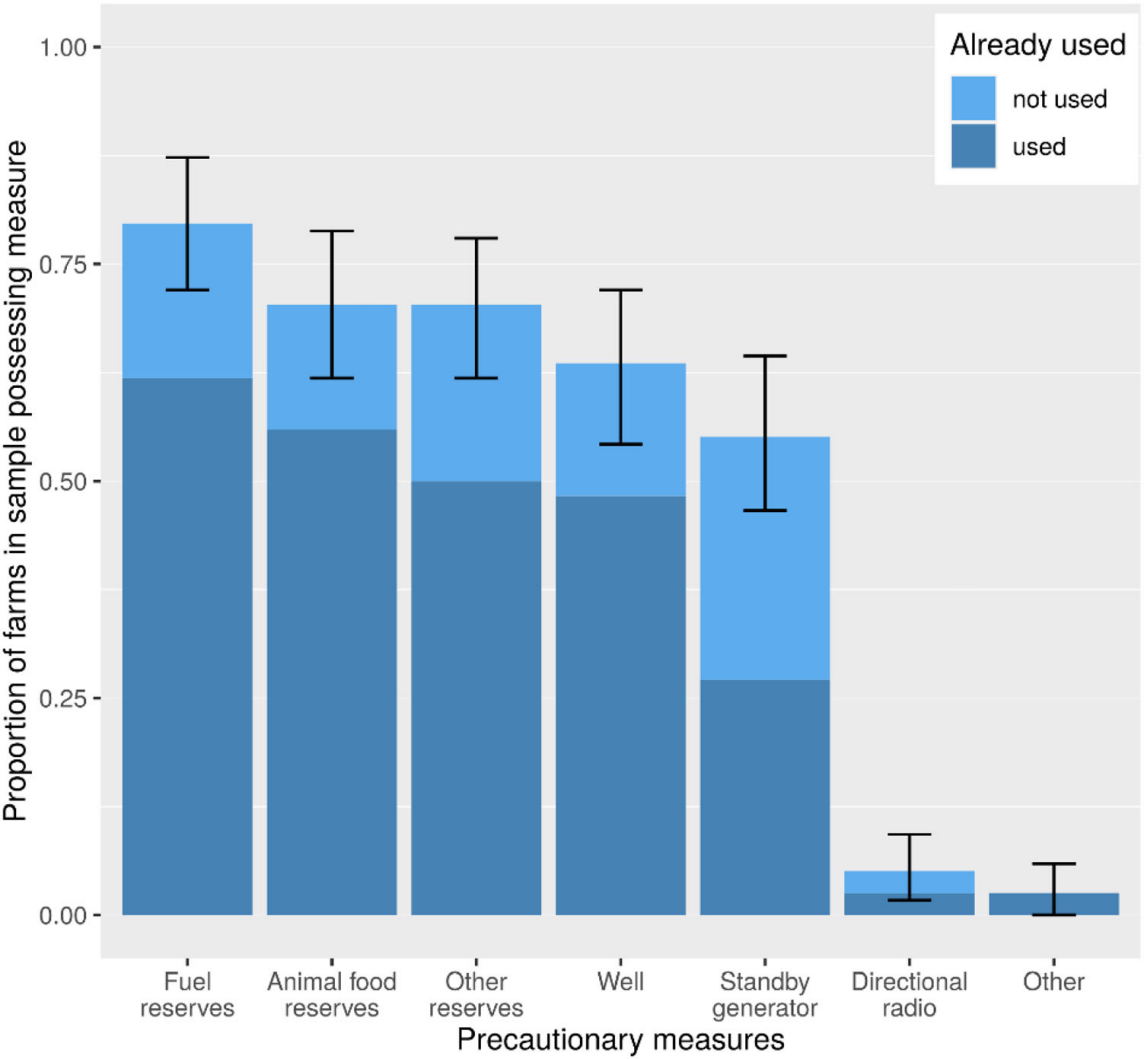
Relative frequency of possession of precautionary measures with bootstrapped 95% confidence intervals. Embedded is information on the percentage of facilities that had to use the precautions in the past

Further, we asked whether the farms possessed different precautionary measures and whether those measures have already been used in the past. The results are depicted in Fig. 4. In contrast to the focus group statements, the share of companies possessing a power generator is unexpectedly low. Just slightly above half of the participants state that their company owns a power generator. This may be a result of the overall stable power grid. As we can see from the data, only about half of those who own a power generator already had the need to use it. Other infrastructures that increase the independence in outage or crisis scenarios (for example, gas reserves) are higher in both possession and need of usage.

**Fig. 5:**
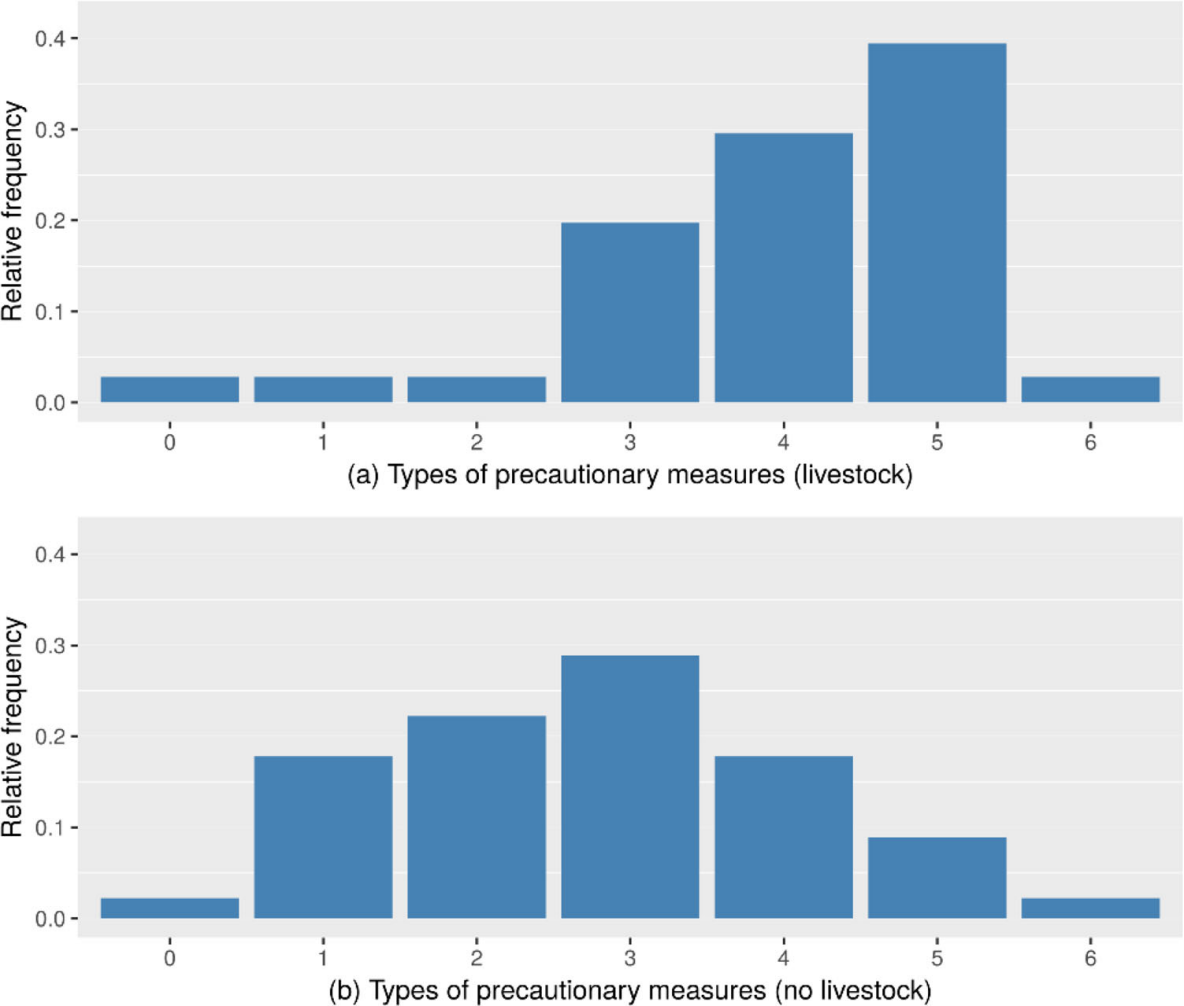
Relative frequencies for the number of different types of precautions for infrastructure failures per farm, with livestock farms in the upper chart (*n*_1_ = 71) and farms without livestock below (*n*_2_ = 47)

Based on the survey data, we are able to test the following hypothesis, which came up in our focus group (Sect. 3.2)—H1 Farms that are active in the livestock sector more often take precautions against outages*.* To test this hypothesis, we compared the number of precautionary measures per farm of farms that did not keep livestock with the ones that did keep livestock. This includes farms that also engaged in other agricultural sectors in addition to livestock. The distributions for farms with or without livestock can be found in Fig. 5. To test the hypothesis H1, we conducted a Wilcoxon rank-sum test. The results indicated a significantly higher level of precautions (*W =* 2417, *p <* 0.001) for farms with livestock (*n*_1_ = 71, *Mdn* = 4) compared to farms not keeping livestock (*n*_2_ = 47, *Mdn* = 3), thus, the results confirm our hypothesis.

#### Estimation of Maintaining Operability

Similar to the focus groups, we also asked the interviewees to estimate the duration of maintaining operational capability in case of different infrastructure outages. Results are rather inconclusive. Only electricity has a rather clear result, with 57.6% (68) stating that the time of operability is less than 24 hours, and 14.4% (17) stating that it is one week or longer. In case of an Internet outage, the anticipated problems have fewer operational consequences as expected: 35.6% (42) of participants think that they are operable less than 24 hours, and 18.6% (22) estimate one week or longer.

In the analysis of our focus group data (Sect. 3.2), we also formulated the following hypothesis—H2 The tools used are often dependent on the Internet*.* To test this hypothesis, we asked the survey participants to estimate how much the technology used on their farms depends on the Internet. Answer options and respective distribution of answers were: Not dependent (5.93%), A little dependent (16.95%), Medium dependent (28.81%), Quite dependent (26.27%), Very dependent (20.34%), and Prefer not to say (1.69%). It should be emphasized that 46.61% of the participants stated that the technology used is quite or very dependent on the Internet.

Relative and absolute frequencies of the answers to the question “Which of your digital tools would continue to function without an Internet connection (e.g., due to a communications network disruption), i.e., would continue to function in ‘offline’ mode?” are depicted in Table 5. Not surprisingly, especially the communication platforms are expected to need an active Internet connection. Unfortunately, 36% (17) of the participants using farm management systems expect their systems not to work in offline scenarios.

**Table 5:** Percentage of answers to the question which (digital) tools would continue to function without an Internet connection

Application	Works Off-line (%)			*n*_*i*_
	−	−/+	+	
Communication platforms	72	18	3	82
Other	50	20	10	8
Farm management information system	36	40	21	46
Herd management system	17	25	55	39
Calculation aid	16	28	53	72
Farm machinery with ISOBUS^a^	12	15	69	50
Automatic milking system	0	33	67	9

The last column (*n*_*i*_) displays the total number of owners of the application. Answer options were: Does not work off-line (−)*,* Works off-line with limitations (−/+)*,* Works off-line (+).

^a^ISOBUS (ISO 11783) is a standardized protocol allowing data exchange between machinery, that is, agricultural tractors can exchange data with attached devices

In the case of ICT failures, classical cloud services are not accessible by definition. To get an idea of how frequently cloud storage is used, we asked which digital tools’ data are stored in a cloud. The relative and absolute frequencies of the answers can be found in Table 6. Here we see to some degree an explanation for the previously mentioned high dependency of farm management systems on the Internet, as data of 67% (29) of the farm management system users are stored in a cloud.

**Table 6:** Percentage of answers to the question whether data of an application are stored in a cloud

Application	Data in Cloud (%)		
	Yes	No	*n*_*i*_
Farm management information system	67	33	43
Communication platforms	49	51	81
Herd management system	45	55	40
Automatic milking system	44	56	9
Other	38	62	8
Calculation aid	25	75	69
Farm machinery with ISOBUS^a^	11	89	47

Since not all participants used every system, the total absolute number (*n*_*i*_) of participants varies per row. Missing values were not included within the relative frequencies.

^a^ISOBUS (ISO 11783) is a standardized protocol allowing data exchange between machinery, that is, agricultural tractors can exchange data with attached devices

One problem stated in the focus groups was the incompatibility of some (digital) tools. Among the users of farm management information systems (47), 68.1% (32) affirmed having problems due to incompatibility issues, and 23.4% (11) did not. The participants that already use digital tools (108) replied to the question “Are your data secured by regular on-site backups?” as follows: 72.2% (78) said yes, 22.2% (24) no, and 5.6% (6) did not give an answer.

## Discussion

Sections 3 and 4 present insights from the focus groups and survey, respectively. In the following section, the answers to the questions underlying this article are summarized and discussed.

### Potential Risks of Digital Tools

During power outages, serious problems arise for different types of agricultural systems. Since digital tools necessarily require electrical power, their use results in a dependency on a working power supply for the long run—even if mobile devices like tablets and laptops could bridge a couple of hours using built-in battery capacity. Crop farms may only experience problems regarding office activities, like documentation and billing. But in animal husbandry, farmers may be faced with the downtime of devices that are crucial for the health of their animals, such as ventilation systems and milking robots. Usually, the general power grid is responsible for delivering an uninterrupted power supply. From the interviews and the study, however, we learned that there are recurring power outages for some companies up to three times a year. Whereas the focus groups indicated that most agricultural companies have an emergency power generator as a precaution, this does not seem to be the case for 42% of the participants in the quantitative survey. As the average estimated fuel capacity covers a few days to one week, longer-lasting power outages and broken emergency power generators represent harmful risks, even for those taking precautions. As more battery-powered devices and vehicles are likely to be used in agriculture in the future (Koerhuis 2020; Spykman et al. 2021) as well as with the good application prospects of photovoltaic systems on farms (Friha et al. 2021), the need for large fuel reserves could decrease.

Assessing the consequences due to telecommunication outages is more complex. Looking first at human-to-human communication, we are in line with the survey results of von Hobe and his colleagues (Hobe et al. 2019), namely that the agricultural actors are well-connected, many players know each other personally, and appreciate the personal contact that promises support in times of trouble. But at the same time there is recognition that there is a countervailing trend towards using more digital communication technologies. Accordingly, all focus groups perceived mobile phones and especially messenger apps to be an essential everyday tool for task coordination. Even though this constitutes just one aspect of communication in agricultural systems, the crucial question concerns the importance of this part of the overall communication for the continuity of agricultural operations. In fact, most agricultural systems in our studies do not vitally rely on (wireless) network connections, but we can confirm a trend towards more digital communication technologies and inter-connected devices in agriculture (Ojha et al. 2015), creating greater vulnerabilities to outages in the future.

Another problem can arise when required machine-to-machine communication is done over air channels, like mobile Internet. Surprisingly, such risks were neither mentioned by the farmers themselves nor present in the literature. We see the first reason for this missing awareness in the lack of extensive implementation, possibly due to risk aversion in the adoption of new technologies (Marra et al. 2003), data protection concerns (Linsner et al. 2021), or the partly unreliable mobile data connection in Germany, especially in rural areas (Fig. 3). The second reason may be a missing understanding of the machine-to-machine aspects of already implemented technologies (Cavallo et al. 2014).

Since precautions regarding telecommunication and Internet outages are difficult to take, especially when personally knowing important points of contact that are essential for business continuity seems to offer advantages. The interviewed farmers (both focus groups and questionnaire) do not own stand-alone radio/wireless technology that would allow them to communicate over the air in case of an ICT breakdown. We strongly recommend the implementation of backup mechanisms for new agricultural applications that depend on ICT, since the operational continuity of agricultural processes during infrastructure outages is essential. Such backup mechanisms should allow community users to work in off-line-scenarios, for example, by having the most relevant data always cached on the client side as well as with the application itself. In general, (new) strong dependencies should be avoided where possible.

### Factors that Affect the Resilience Capacities

Based on our qualitative data and quantitative verification, we are able to assess the level of the three resilience capabilities (Meuwissen et al. 2019) for the domain of modern agricultural businesses.

#### Robustness

In case of failures in the energy or communication infrastructures, almost all companies are unable to contain disruptions. While livestock farming has a great need for electricity, arable farming is rather independent of electricity but has high demands on a functioning communication infrastructure. Disturbances in both infrastructures, electricity and communication, are hard to absorb, especially for small and medium enterprises. Solutions that absorb power grid outages are available, for example, in the form of microgrids or decentralized energy systems (Panteli and Mancarella 2015), although typically these are not considered by small companies. Rare exceptions are self-sustaining companies with photovoltaic and/or wind power stations, which allow the absorption of general power grid outages. ICT failures in the form of Internet service breakdowns are hard to absorb, since required technology changes are currently not available to customers. Especially agricultural businesses in rural areas usually do not have a consistent Internet connection due to frequent gaps in broadband and mobile Internet coverage. About 35.6% of the respondents (Sect. 4.2.2) think they can only remain operable for less than 24 hours in case of an Internet outage. In the future, communication network redundancy could be established by affordable orbital Internet connections (Harris 2018) or the use of wireless sensor networks for communication in disaster scenarios (Adeel et al. 2019) to further improve the technical resilience of agricultural companies.

#### Adaptability

Most of the surveyed companies own power generators—either voluntarily or required by law. Because agricultural machinery depends on fuel, a sufficient stock of fuel is expected to be available in most companies to fuel the power generators for multiple days on average. The effort and time to put a power generator into operation range from “easy”/“quick” to “enormous”/“couple of hours” (both statements in fg3), depending on the company’s system construction and the technologies involved. In total, the adaptability capacity regarding power outages is high due to the ability to completely restore full operation after a power outage for a limited time. Adaption of communication can also be realized by switching from phone-based communication to face-to-face conversations. These personal conversations are locally possible and often preferred by well-networked farmers (Fecke et al. 2018; Hobe et al. 2019).

#### Transformability

When the regular power grid returns to normal operation after an outage, companies just have to disconnect the temporarily installed power generators. In some cases, this process requires modern digital equipment and time-consuming actions to reset the whole agricultural system into a fully operational mode. Looking at communication infrastructure, there was no required effort mentioned for restoration into regular operational mode. This may be because these devices will typically reconnect automatically after the recovery of the ICT infrastructure. In such a case, the regular way of communicating via telephone, email, or similar digital options is working again without the need for active interventions.

### Limitations

It has to be considered that our sample for the focus groups and the questionnaire consisted only of farmers. Other possible stakeholders, such as their business partners or people working on agricultural ICT, were not involved. The participants in the focus groups run their agrarian businesses in southwestern Germany, consisting of small structured areas, which is not representative for the whole country (BMEL 2018). In contrast, with the quantitative study (questionnaire), we had participants from all parts of Germany.

Moreover, the familiarity with and expertise in agricultural digital technologies of our samples may be biased. Most of the participants in focus groups took part in a federal education initiative that incorporates digital tools for farming businesses as a subject.

As is the case with all mixed methods approaches, we decided for a trade-off between the detailed, subjective information of qualitative statements against quantitatively measurable dimensions. The individual results of the sub-studies should therefore only be interpreted collectively, as the hypotheses generated in Sect. 3 and their verification in Sect. 4 successively build on each other.

## Conclusion

As mentioned by related literature, digitalization can increase the efficiency and effectiveness of agricultural operations (Walter et al. 2017; Wolfert et al. 2017). Trends also point to an increasing use of digital services in this area (Sundmaeker et al. 2016; Fecke et al. 2018). To critically challenge the predominantly tech-positive body of literature on digitalization in agricultural systems, we looked at the specific risks of digital tools for farm systems and their impact on resilience capacities. This is inevitable in terms of the development of sustainable and future-proof systems. For this purpose, we used a mixed methods approach to first generate hypotheses through qualitative focus group interviews (*N* = 52), which we verified in a second step through quantitative questionnaire surveys (*N* = 118). To gain insight into dependence on digital technologies, we asked explicitly about aspects of digitalization on household-level agricultural systems, but also inquired about risks and disasters experienced, as well as explored precautions taken. Key insights about potential risks associated with the use of digital tools (RQ1) are as follows:

- The incorporated tools of agricultural operations are often dependent on the Internet.
- A power failure of a few hours to a few days would not be very harmful to those farms having a power generator. Longer failures would realistically result in complete harvest failures and lethal consequences for animals.

Key findings related to resilience capacities (RQ2) are as follows:

- Most of the farmers are not aware of how the Internet and mobile network infrastructure affect their agricultural system and do not take any precautions for ICT breakdowns.
- Just slightly above half of the farmers own an emergency power generator.
- Farms that are active in the livestock sector more often take precautions against outages than do other farmers, both for higher dependency on continuous-working machinery and because of legal requirements (Sect. 3.2.2).

Especially problematic for farms (Fig. 3) is the trend of increasing dependency on Internet infrastructure along with the fragility of the Internet. Additionally, the statements by interviewed farmers show the diversity in the usage of digital tools. Some farmers are limited in the usage of digital tools by external factors, like high investment costs, missing information on these modern farming tools in their education and experience, or a bad/missing Internet connection in their region.

Our assessment regarding resilience capacities with a focus on the two infrastructures of electric supply and communication shows that there are high robustness and adaptability capacities in these systems, but that transformability capacity is low due to mainly technical reasons and should therefore be of particular interest for future work. When looking at the digitalization of the agricultural sector, it is important to keep in mind that all advantages must go hand in hand with strong operational reliability. Currently, there is no publication investigating the operational reliability of present tools, nor are there descriptions of technical approaches for resilient communication that would allow strengthening the robustness capacity of farming companies. More research in this field is needed. Market analyses inspecting digital products for agriculture with regard to resilience criteria would allow for a more precise assessment of the current situation. Also, research on utilizing the increasingly distributing Internet of Things networks for self-operated communication between neighbored farms could help to achieve greater resilience capacities.
